# Carbon Monoxide Gas Is Not Inert, but Global, in Its Consequences for Bacterial Gene Expression, Iron Acquisition, and Antibiotic Resistance

**DOI:** 10.1089/ars.2015.6501

**Published:** 2016-06-10

**Authors:** Lauren K. Wareham, Ronald Begg, Helen E. Jesse, Johan W.A. van Beilen, Salar Ali, Dimitri Svistunenko, Samantha McLean, Klaas J. Hellingwerf, Guido Sanguinetti, Robert K. Poole

**Affiliations:** ^1^ Department of Molecular Biology and Biotechnology, The University of Sheffield, Sheffield, United Kingdom.; ^2^ School of Informatics, The University of Edinburgh, Edinburgh, United Kingdom.; ^3^ Molecular Microbial Physiology, Swammerdam Institute for Life Sciences, University of Amsterdam, Amsterdam, The Netherlands.; ^4^ Biomedical EPR Facility, School of Biological Sciences, University of Essex, Colchester, United Kingdom.

## Abstract

***Aims:*** Carbon monoxide is a respiratory poison and gaseous signaling molecule. Although CO-releasing molecules (CORMs) deliver CO with temporal and spatial specificity in mammals, and are proven antimicrobial agents, we do not understand the modes of CO toxicity. Our aim was to explore the impact of CO gas *per se*, without intervention of CORMs, on bacterial physiology and gene expression. ***Results:*** We used tightly controlled chemostat conditions and integrated transcriptomic datasets with statistical modeling to reveal the global effects of CO. CO is known to inhibit bacterial respiration, and we found expression of genes encoding energy-transducing pathways to be significantly affected *via* the global regulators, Fnr, Arc, and PdhR. Aerobically, ArcA—the response regulator—is transiently phosphorylated and pyruvate accumulates, mimicking anaerobiosis. Genes implicated in iron acquisition, and the metabolism of sulfur amino acids and arginine, are all perturbed. The global iron-related changes, confirmed by modulation of activity of the transcription factor Fur, may underlie enhanced siderophore excretion, diminished intracellular iron pools, and the sensitivity of CO-challenged bacteria to metal chelators. Although CO gas (unlike H_2_S and NO) offers little protection from antibiotics, a ruthenium CORM is a potent adjuvant of antibiotic activity. ***Innovation:*** This is the first detailed exploration of global bacterial responses to CO, revealing unexpected targets with implications for employing CORMs therapeutically. ***Conclusion:*** This work reveals the complexity of bacterial responses to CO and provides a basis for understanding the impacts of CO from CORMs, heme oxygenase activity, or environmental sources. *Antioxid. Redox Signal*. 24, 1013–1028.

## Introduction

CO is a notoriously toxic gas, binding primarily to ferrous oxygen-reactive heme proteins (21). Nevertheless, CO is generated endogenously in mammals by heme oxygenase (HO)-catalyzed breakdown of heme (62), and, although relatively inert from a chemical standpoint, it is a gasotransmitter or small gaseous signaling molecule with critical biological activities, including vasodilation and anti-inflammatory mediation (47).

The role of CO in pathogenesis and host defense against microbial infection is obscure, with conflicting and unexpected roles reported [reviewed in Wareham *et al.* (70)]. For example, Wegiel *et al.* recently hypothesized that bacteria exposed to CO release ATP, which activates inflammatory pathways (71). Although CO may be toxic toward microorganisms [being used to preserve meat (52)], many bacteria are relatively resistant, in part, because they possess CO-insensitive oxidases, such as cytochrome *bd* (32). Indeed, airborne bacteria survive high urban CO concentrations (39), and bacterial cultures may be bubbled with CO with little toxicity (71).

Innovation The beneficial effects of CO-releasing compounds (CORMs) in physiological and antimicrobial therapies are generally attributed to CO, yet bacteria tolerate this gas. This is the first analysis of the global impact of CO (without a CORM) on bacterial growth, gene expression, and responses to stress, thus underpinning interpretation of studies that employ CORMs. Tightly controlled chemostat growth and statistical modeling show that not only global transcriptional responses occur in energy metabolism but also iron transport and thus metal chelator sensitivity and the metabolism of arginine and sulfur amino acids. Unlike other gasotransmitters (H_2_S and NO), CO provides negligible protection against antibiotics.

CO-releasing molecules (CORMs) were developed for temporal and spatial CO delivery in therapy without intoxication. CORMs are generally metal carbonyls with one or more labile CO groups, which are released by ligand exchange reactions, enzymatic activation, or photoactivation [references in Wareham *et al.* (70)]. Many CORMs have potent antimicrobial effects, but the mechanism of toxicity has been debated. Even the significance of CO release is unclear, although CORM-derived CO does target oxidases *in vivo* and CORMs elicit multiple transcriptomic changes in respiratory gene expression (41). Since the actions of CORMs are distinct from antibiotics (70), they are promising replacements for, or alternatively adjuvants to, conventional overused antibiotics in fighting antibiotic-resistant strains. The antibiotic-potentiating effects of certain CORMs (65) have not been reported for CO gas, even though NO and H_2_S confer some defense against antibiotics (24, 59).

In this study, we present the first systematic multilevel analysis of the bacterial effects of CO gas. Transcription factor (TF) measurements and modeling reveal that gene expression is highly perturbed with major consequences for energy metabolism, iron homeostasis, and amino acid metabolism. Interestingly, a CORM, but not CO gas, is an effective adjuvant to antibiotics, highlighting the importance of the metal ion in bacterial toxicity.

## Results

### Growth of *Escherichia coli* aerobically and anoxically in the presence of CO gas

Apart from carboxydobacteria, which oxidize CO to CO_2_, little is known about the effects of CO on growth of bacteria. To establish a sublethal concentration of CO for analyses, *E. coli* cells were grown in a batch bioreactor in Evans medium (41) with glucose. In the mid-log phase, the gas mix was switched to 50% CO (by volume, 100 ml·min^−1^). CO only slightly inhibited growth aerobically (Supplementary Fig. S1A; Supplementary Data are available online at www.liebertpub.com/ars): growth was linear (not exponential) and the doubling time at the point of CO addition was about 1.6 h, increasing to 2.2 h with CO. Anoxically, however (Supplementary Fig. S1B), CO inhibited growth and a lower biomass was attained.

### Overview of transcriptomic responses to CO

**FIG. 1.**
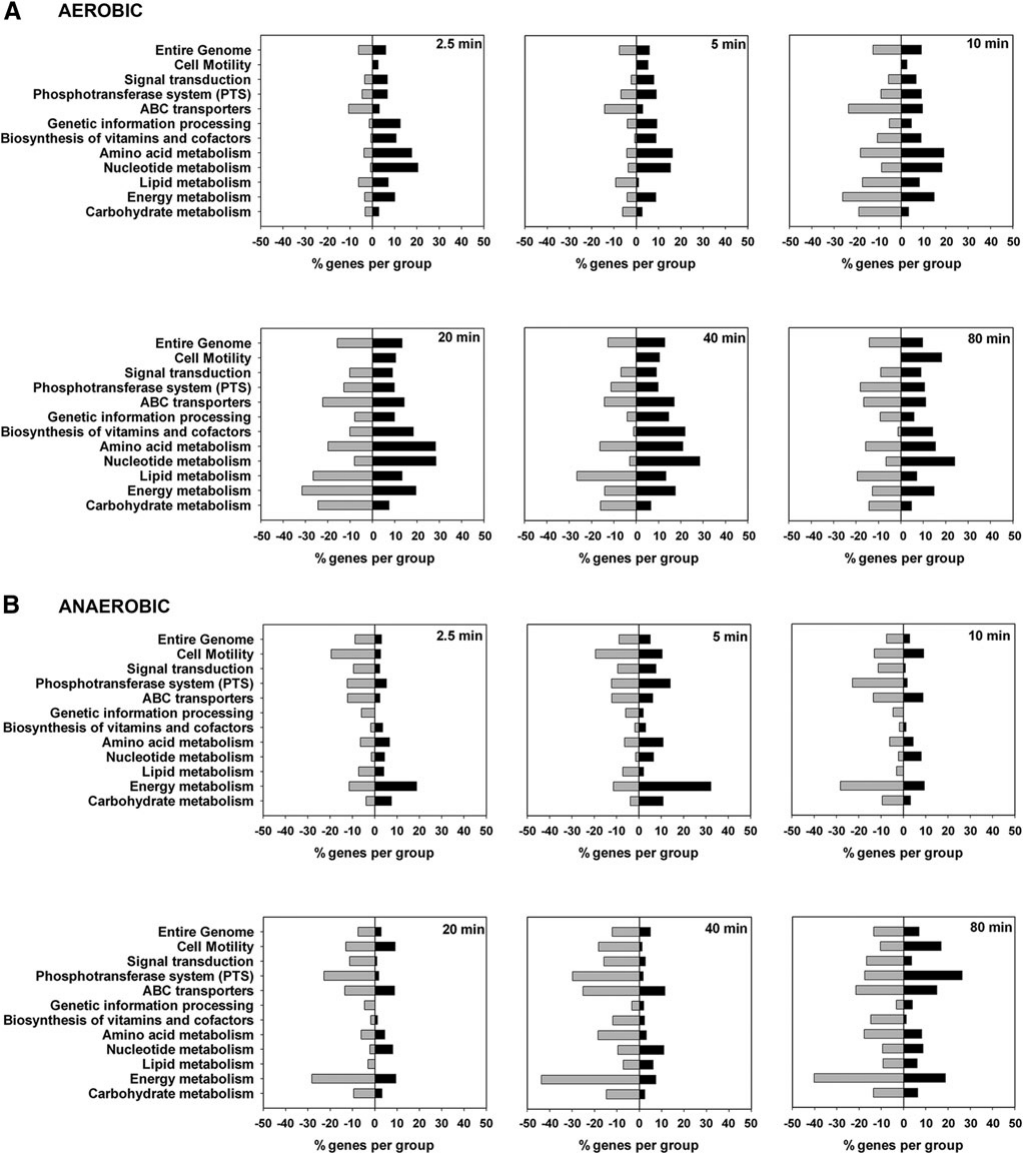
**Functional categories of genes affected by CO gas addition in aerobic and anaerobic conditions.** Transcriptomic analyses were performed at 2.5, 5, 10, 20, 40, and 80 min after the flow of CO gas was initiated in both aerobic **(A)** and anaerobic **(B)** conditions as described in the Materials and Methods section. Genes are grouped according to functional categories. The percentages of genes in each panel that showed elevated expression (*black bars*, *right*) or reduced expression (*gray bars*, *left*) are shown.

To determine how cells respond globally to CO, we conducted transcriptomic analyses on samples taken immediately before CO gas addition and at regular timed intervals thereafter. CO altered expression of numerous genes, defined as ≥2-fold up or ≥2-fold down (the latter representing a change of ≥0.5 of the control transcript level). Aerobically, exposure to CO gas for 20 min led to 29% of the genome significantly changing, decreasing to 24% after 80 min. Anoxically, most significant gene changes were seen only after 80 min of CO exposure, where 20% of the genome changed significantly in expression (Fig. 1).

### Aerobic and anaerobic treatments with CO perturb central energy metabolism

CO competes with oxygen for oxidases, inhibiting aerobic respiration. Classically, a CO:O_2_ ratio of 4:1 (11) is used to measure CO inhibition of respiration and its photochemical relief; thus, the transcriptomic responses to CO of genes involved in energy metabolism were of special interest. In facultative bacteria, the tricarboxylic acid (TCA; Krebs) cycle and glycolysis generate reducing equivalents that feed into multiple oxygen (or other oxidant)-terminated respiratory chains that conserve energy as the protonmotive force for ATP synthesis and membrane transport. Maximal transcript changes occurred after 20 min aerobically [52% of 149 genes in this category; 32% of genes were downregulated and 20% were upregulated (Fig. 1A)].

**FIG. 2.**
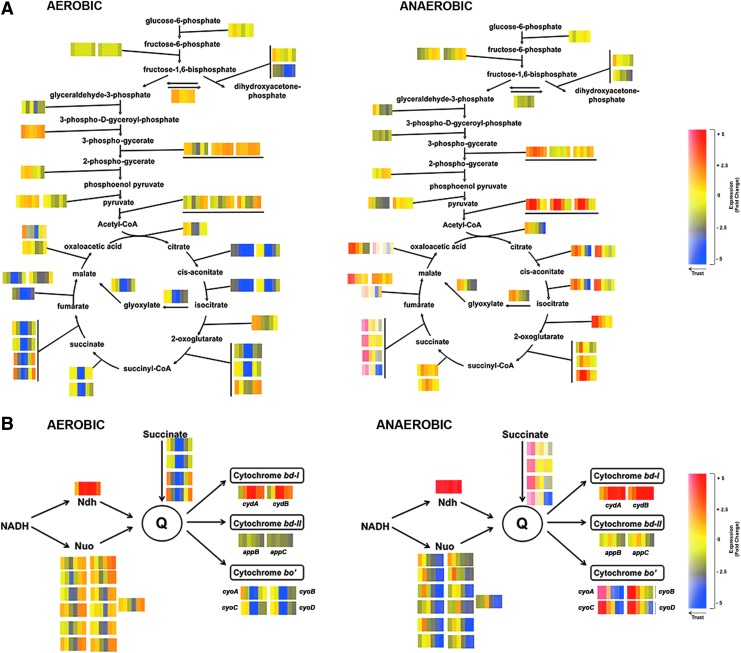
**CO-induced transcriptomic changes of genes involved in the TCA (Krebs) cycle, glycolytic pathways, and O_2_-dependent electron transport pathways in aerobic and anaerobic conditions. (A)** The major routes of carbon flow through the Embden–Meyerhof pathway and Krebs cycle are shown. **(B)** Reducing equivalents from NADH are fed *via* two NADH dehydrogenases (Ndh, Nuo) to a quinone pool (Q) and thence to one of three terminal oxidases (cytochromes *bd*-I, *bd*-II, and *bo*′). Each block of color strips indicates a single gene involved in the reaction step shown and, within each block, the vertical strips show (from *left* to *right*) changes in gene expression at the sampling points (2.5, 5, 10, 20, 40, and 80 min after introducing CO gas). Changes in gene expression are illustrated by the heat map (*right*): *blue color* indicates a gene that is downregulated, *red color* indicates upregulation, and *yellow color* indicates no change in transcriptomic level. TCA, tricarboxylic acid. To see this illustration in color, the reader is referred to the web version of this article at www.liebertpub.com/ars

Figure 2 illustrates pathways of aerobic and anaerobic central metabolism and the CO-induced changes in expression of genes that encode each step. Each color block (separated into six vertical strips) represents a single gene; the strips from left to right in each block denote the transcript level, by reference to the heat scales, at each time increment in the experiment, that is, 2.5, 5, 10, 20, 40, and 80 min after CO treatment (as in Fig. 1). For example, the conversion of acetyl-CoA to citrate is mediated by *gltA* that encodes citrate synthase (Fig. 2A, left). The adjacent color block indicates that aerobically it is transiently downregulated (blue).

Aerobically, CO caused little change in genes involved in glycolysis, but transient downregulation of most Krebs cycle genes, including aconitase (catalyzing citrate to isocitrate *via* cis-aconitate; *acnAB*); α-oxoglutarate dehydrogenase; succinyl-CoA synthetase (α-oxoglutarate to succinyl-CoA; *sucAB*); lipoamide dehydrogenase [the *lpd* gene product, E3 component of three multicomponent enzyme complexes (16)]; succinate dehydrogenase (succinate to fumarate; *sdhABCD*); and fumarase (fumarate to malate; *fumABC*) (Supplementary Fig. S2A for fold changes and details of relevant TFs). The downregulation of Krebs cycle genes in aerobic conditions may reflect a backlog of reducing equivalents on respiratory inhibition by CO.

In contrast (Fig. 2A, right and Supplementary Fig. S2A), anaerobically, CO upregulated certain genes of the Krebs cycle, in particular genes encoding pyruvate dehydrogenase (PDH; *aceEF, lpd*), aconitase (*acnAB*), and succinate dehydrogenase (*sdh*) (Supplementary Fig. S2A), with smaller changes in glycolysis. The anaerobic response was immediate: after 5 min of CO bubbling, *aceEF* and *lpd* (together encoding the PDH complex) were upregulated 7-fold and 5-fold, respectively. PDH generates acetyl CoA and acts as a gateway for flux of metabolites into the Krebs cycle. Most highly upregulated after 2.5 min, by up to 13-fold, were the succinate dehydrogenase genes (*sdhABCD*), followed by a 10-fold downregulation of *sdhD* after 40 min.

### Consequences of CO exposure for components of the respiratory chain

CO inhibits aerobic respiration by binding to all three terminal oxidases (32). Oxidase structural genes are under complex transcriptional control, primarily by oxygen (55), but there are no reports of CO effects. However, aerobically (Fig. 2B, left), there was a 5- to 10-fold decrease in expression of the *cyo* operon (*cyoABCD*) that encodes the cytochrome *bo*′ (*bo*_3_) oxidase, but an increase in *cydAB* expression, which encodes cytochrome *bd-I* (4-fold upregulation after 20 min) (Supplementary Fig. S2B shows fold changes and relevant TFs). This is consistent with the properties of the oxidases: cytochrome *bd*-I has high oxygen affinity (19) and a stoichiometry of 1 H^+^/electron, whereas cytochrome *bo*′ has lower oxygen affinity and a stoichiometry of 2 H^+^/electrons (18, 57). Interestingly, cytochrome *bd* is also less sensitive than cytochrome *bo*′ to other respiratory inhibitors such as cyanide (3), azide (50), and NO (40). Aerobically, changes in expression of the enigmatic cytochrome *bd*-II encoded by *appBC* were slight. In the presence of CO, electron flow into the aerobic transport chain is facilitated in both aerobic and anoxic conditions by upregulation of NADH dehydrogenase (*ndh*), whereas succinate dehydrogenase (*sdhABCD*) transcripts diminished (Fig. 2B, left and Supplementary Fig. S2A).

Major changes in terminal oxidase genes were not expected anaerobically due to the absence of oxygen or alternative respiratory electron acceptors. However, the *cyoABCD* genes responded dramatically to CO by upregulation of the entire operon by 20- to 30-fold after only 5 min of CO exposure (Fig. 2B, right and Supplementary Fig. S2B). The response dampened after 40 min. The effects of CO on *ndh* and *cydAB* echoed the response aerobically (Fig. 2B, left and right), but the responses anoxically were greater, with *ndh* maximally expressed at 80 min; however, in contrast to aerobic conditions, the *nuo* genes were uniformly downregulated (2- to 5-fold) after 40 min with CO (Fig. 2B, right).

We also assayed the levels of the various quinone species (labeled Q in Fig. 2B) that act as membrane-bound electron and proton carriers from dehydrogenases to the oxidases. Although *E. coli* responds to reduced oxygen availability on a timescale of seconds, with synthesis (under post-translational control) of quinones involved in anaerobic respiration (7), we found no significant changes in the pool sizes of ubiquinone (UQ), menaquinone (MK), or demethylmenaquinone (DMK) on CO challenge (Supplementary Fig. S3).

Finally, certain CORMs cause accumulation of ROS (66). However, inspection of the complete data sets (which can be consulted at GEO, see the Materials and Methods section) reveals that oxidative stress genes (*sodA, sodB, sodC, katE, katG, oxyR, oxyS*) were not upregulated by CO gas, either aerobically or anaerobically. This suggests that CO gas does not induce oxidative stress.

### Modeling of the transcriptomic data by TFInfer

The abundance of TFs that underlie multiple fluctuations in transcript levels frustrates data interpretation. In this study, we used the TFInfer approach (4, 37), a Bayesian statistical method that integrates gene expression data with regulon information (culled from Regulon DB or EcoCyc) to identify TF activity profiles that aid in understanding of the raw transcriptional changes. To compare results from TFInfer on different data sets (CO gas under aerobic and anoxic conditions), we used intuitive coherence plots that highlight differences in the magnitude and kinetics of the response to the two stimuli.

**FIG. 3.**
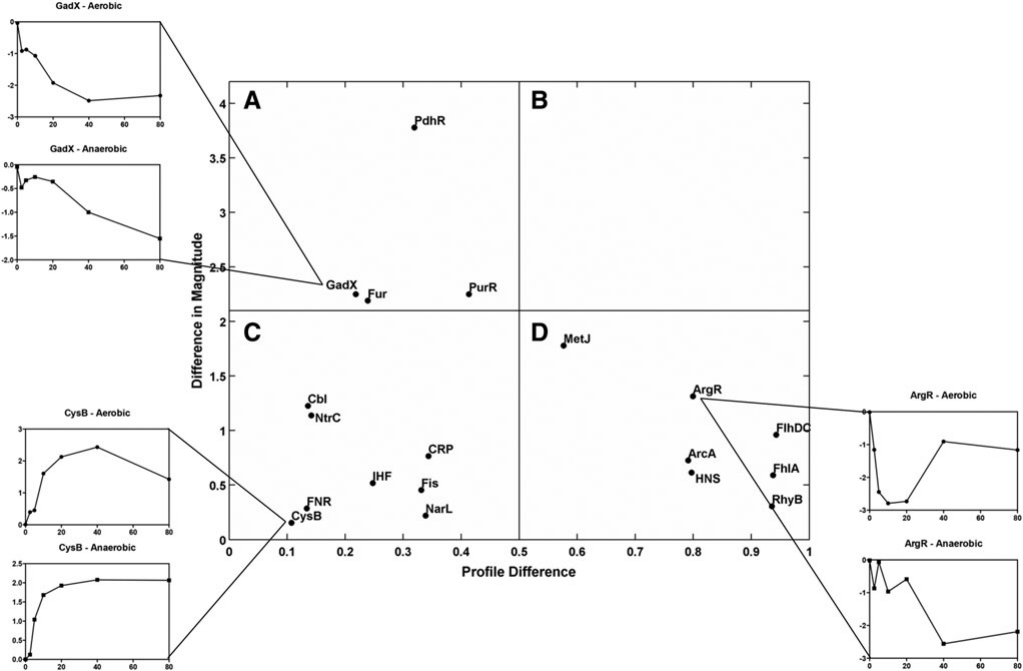
**TFInfer correlation profile of TF activities in CO aerobic conditions** ***versus*** **anoxic conditions.** Profile differences are plotted on the abscissa and differences in magnitude on the ordinate. In quadrants **(A, C, D)**, the dynamics of one representative TF are shown both aerobically and anaerobically. For example, the response profiles for the TFs, FNR and CysB, are similar in both conditions, in both the magnitude of the response and their correlation (appearing in the *lower left quadrant*), while GadX and PdhR (*upper left*) have a similar response in terms of the shape of the profile, while the magnitude of the response is greater in anaerobic conditions than aerobic conditions. On the other hand, the response of the TF MetJ is different both in magnitude and in the shape of the profile, lying near the interface of the upper and *lower right quadrants*. ArgR and HNS have a similar response in terms of the magnitude of the profile, while the shape of the activity curve of the response is greater in aerobic conditions than anaerobic conditions. Absolute Pearson correlations in the *middle* indicate only weak similarity between profiles. FNR, fumarate nitrate reduction regulator; TF, transcription factor.

Figure 3 summarizes the differences between two sets of TFInfer data (growth with CO either aerobically or anoxically) in a scatter plot. The x-coordinate of each point (labeled with the TF identity) represents the profile difference between the two conditions, computed as 1 minus the absolute Pearson correlation coefficient between the two profiles, while the y-coordinate represents the change in magnitude of the response (computed as the absolute difference of the norms of the two profiles). Hence, TFs whose response is similar both in magnitude and kinetics are located near the origin of the coherence plot, while TFs in the top right corner of the plot respond differently in both kinetics and amplitude. In quadrants A, C, and D, one representative TF is shown to illustrate the value of the plots. Error bars (shown only in Supplementary Fig. S4) represent 95% confidence intervals; to reduce clutter, we have omitted TFs where the error bars for the absolute Pearson correlation were greater than ±0.15.

For example, under acid conditions, GadX (with GadE) positively regulates *gadA* and *gadBC*, which encode components of the glutamate-dependent acid response (68). GadX is inducible by NO (9), but was not known to be regulated by CO. While the magnitude of the GadX response is different aerobically and anaerobically (quadrant A), that of CysB is similar in both conditions, reflected in its location in quadrant C of Figure 3. Its role in cysteine metabolism is discussed below. Finally, ArgR in quadrant D shows marked profile differences aerobically and anaerobically; its role in arginine metabolism is also explained below.

### The inferred role of ArcA in the response of *E. coli* to CO

**FIG. 4.**
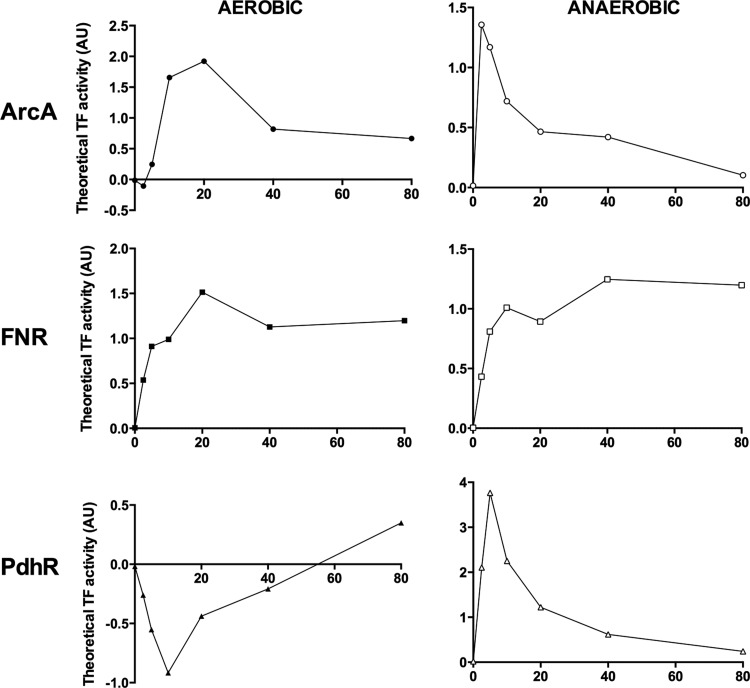
**Inferred activity of the TFs, ArcA, FNR, and PdhR, in response to CO.** Response profiles for ArcA (*top*, *circles*), FNR (*middle*, *squares*), and PdhR (*bottom*, *triangles*) are shown as predicted by TFInfer. TF activity is plotted for each time point using TFInfer data modeled on gene changes in response to CO at each time point. Aerobic profiles are shown with closed symbols (*left*) and anoxic profiles with open symbols (*right*).

The TFInfer coherence plot reveals three TFs that collectively regulate genes in central metabolism: PdhR (Fig. 3A), fumarate nitrate reduction regulator (FNR) (Fig. 3C), and ArcA (Fig. 3D). Each TF responds distinctively to CO. ArcA responded differently under each condition (Figs. 3D and 4). ArcAB, a two-component system, indirectly senses oxygen *via* the redox state of the quinone pool, fermentation products, and perhaps other factors (2). Under anoxic or microaerobic conditions, ArcB autophosphorylates, then transphosphorylates ArcA through a phosphorelay, thereby increasing the affinity of ArcA for its DNA targets (31). Phosphorylated active ArcA (ArcA-P) then represses expression of genes involved in respiration (*e.g.,* electron transport enzymes, cytochrome *bo*′, and the Krebs cycle enzymes) and activates genes involved in fermentative metabolism and cytochrome *bd.* Thus, in a mutant lacking two of the three oxidases, the aerobic expression of ArcA-P-activatable genes, such as *cydAB*, is elevated, but that of ArcA-P-repressible genes, such as *cyoABCDE*, is lowered (30) because the quinone pool is trapped in a reduced form and unable to inhibit the autokinase activity of ArcB. CO would be expected to mimic such an absence of oxidase activity by inhibiting electron transfer to oxygen.

The modeled changes in ArcA activity (Fig. 4) are consistent with this: aerobically, ArcA activity increases rapidly, in part, due to quinone over-reduction. After 20 min, declining ArcA reflects partial reversal of the effects of CO. This pattern may relate to a transient overshoot of the UQH_2_/UQ ratio observed in transitions from aerobic to sustained anaerobic conditions [(7); JWA van Beilen and KJ Hellingwerf, unpublished]. The increased activity of ArcA represses Krebs cycle gene expression in the first 20 min of CO exposure (Fig. 2A), but enhances *cydAB* expression (Fig. 2A, B). Thus cells respond, not directly to CO, but to diminished oxygen availability due to competition by CO.

Anoxically, the falling ArcA(-P) activity correlates with the increase in expression of Krebs cycle and *cyo* genes (Fig. 2A, right) (55). This may perhaps be attributed to inhibition by fermentation products of ArcB phosphatase activity (29, 31) rather than the respiratory quinone pools since these would not be expected to display redox changes under anoxia in the absence of alternative electron acceptors (6, 55). Anoxic CO exposure increased cytochrome *bd*-1 gene expression (Fig. 2B), as expected from the initial rise in ArcA activity.

### Phosphorylation of ArcA and pyruvate accumulation in the response to CO

**FIG. 5.**
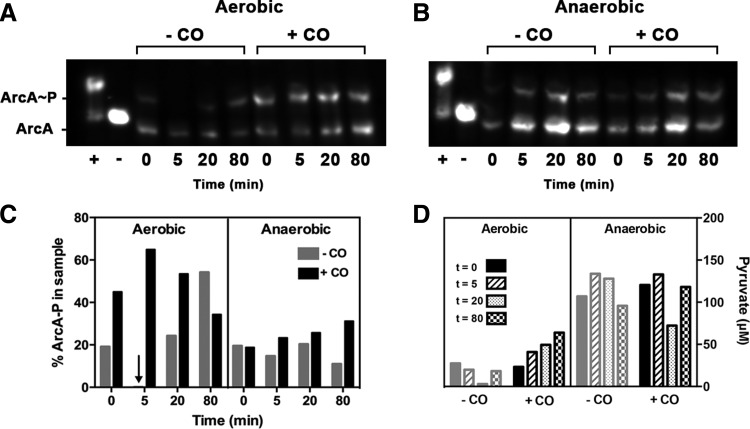
**The phosphorylation state of ArcA in the absence and presence of CO and corresponding pyruvate levels in cells. (A, B)** Typical Western blots developed using ArcA polyclonal antiserum for Phos-tag™ gels and cultures grown with/without CO at the time points shown, both aerobically and anaerobically. Controls with purified ArcA are shown: the lane labeled “+” shows ArcA-P (25 ng purified ArcA phosphorylated *in vitro* using carbamoyl phosphate), and the lane labeled “−” shows 25 ng unphosphorylated ArcA. **(C)** The percentage of Arc-P in each sample was calculated from band intensities; results show ArcA-P expressed as a percentage of total ArcA/ArcA-P in each sample.**(D)** Pyruvate levels were assayed in culture supernatants collected over time (in each condition: *solid bars*, t = 0; *hashed bars*, t = 5; *spotted bars*, t = 20; *checkered bars*, t = 80).

The role of Arc in the CO response was confirmed by direct assay of the phosphorylation state of ArcA (Fig. 5A–C). To measure the proportion of phosphorylated ArcA as a percentage of the total protein, Phos-tag™-acrylamide gel electrophoresis was used, followed by Western blotting. Within 5 min of aerobic CO exposure, and persisting for >20 min, there was a marked increase in ArcA-P, consistent with the inferred ArcA activity profile (Fig. 4). Anaerobically, significant levels of ArcA-P were present at 5–80 min, irrespective of the presence of CO (Fig. 5C), also consistent with ArcA activity (Fig. 4). Precise temporal correlation is impractical given the limitations of sampling.

The effect of fermentation products, such as lactate and pyruvate, on ArcB is obscure, but D-lactate, pyruvate, and NADH enhance the autophosphorylation of ArcB (29). We therefore assayed extracellular pyruvate and showed (Fig. 5D) that (over 80 min) CO markedly increases aerobic accumulation of pyruvate (67). Anaerobically, as expected, pyruvate accumulated to higher levels, irrespective of the presence of CO. Interestingly, PdhR activity (Fig. 4) declined during aerobic accumulation of pyruvate (Fig. 5).

### The role of FNR in the response of *E. coli* to CO

**FIG. 6.**
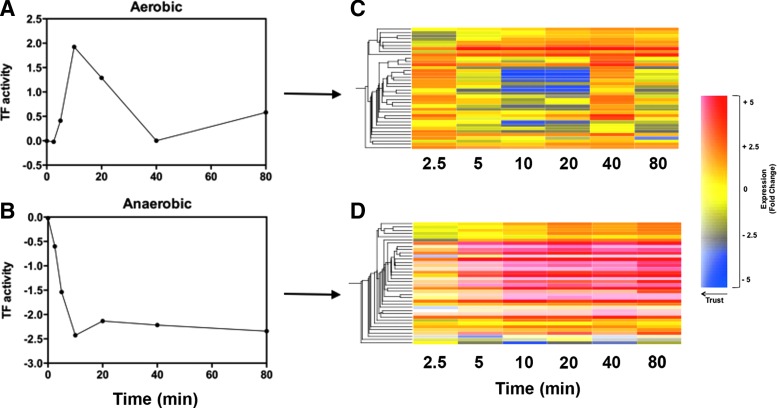
**Predicted TF activity of Fur, a transcriptional repressor of Fe-regulated genes, and corresponding gene changes.** TFInfer data over time for Fur are shown under **(A)** aerobic and **(B)** anaerobic conditions in the presence of CO. **(C)** and **(D)** map changes in iron-regulated genes aerobically and anaerobically, respectively. *Yellow* indicates genes that remain unchanged in the presence of CO, *red* indicates genes upregulated, and *blue* indicates genes downregulated, according to the heat map (*right*). The intensity of the color is indicative of the trust placed on that measured level of regulation after three biological repeats. Feature extraction from the scanned arrays and subsequent data analysis used GeneSpring GX v7.3. The clustering at the *left* is a GeneSpring gene tree and shows genes organized by their similarity in response to the imposed conditions. To see this illustration in color, the reader is referred to the web version of this article at www.liebertpub.com/ars

**FIG. 7.**
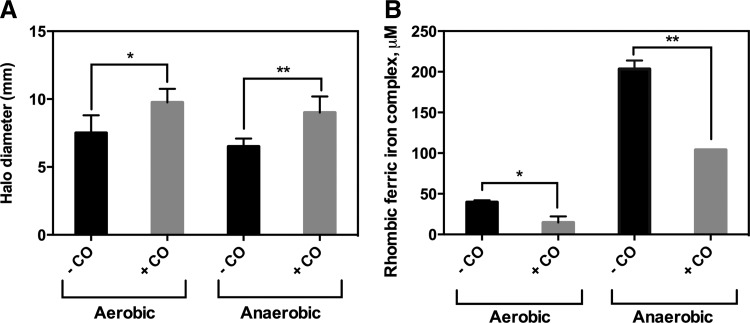
**Cells exposed to CO produce higher levels of siderophores as free intracellular iron pools are depleted.** Stationary *Escherichia coli* cultures were grown and 10 μl aliquots of culture were spotted onto 25-ml CAS agar plates. Cells were incubated in aerobic (air)/aerobic + CO (air +50% CO) or anaerobic (N_2_)/anaerobic + CO (N_2_/50% CO) conditions for 24 h. The diameter of the cells plus halo and the diameter of the cells alone were measured and the difference plotted in **(A)**. The presence of CO increased siderophore production in aerobic and anaerobic conditions; results are plotted as mean ± SD of two biological repeats each with four technical replicates. A paired *t*-test shows *p of 0.03 and **p of 0.008. **(B)** The ferric iron in rhombic coordination in whole cells as measured by electron paramagnetic resonance (EPR) spectroscopy shows that the iron levels decreased both aerobically and anaerobically on exposure to CO gas. Results are plotted as mean ± SD of two biological repeats each with two technical replicates. A paired *t*-test shows *p of 0.05 and **p of 0.005. CAS, Chrome Azurol S.

The ligand-reactive FeS clusters of FNR make it a direct sensor of oxygen availability. Under microaerobic/anaerobic conditions, it negatively regulates Krebs cycle (*e.g., fumA*, *sdhABCD*, *sucABCD*) and oxidase genes (*cydAB* and *cyo* operons) (14). TFInfer (Fig. 4) predicted that FNR activity increases in the presence or absence of oxygen after CO treatment. Nevertheless, the *cydAB* operon was also upregulated after CO exposure in both conditions (Fig. 2B and Supplementary Fig. S2B), implicating additional factors. Indeed, the *cydAB* operon is under complex regulation, being repressed by FNR and the nucleoid DNA-binding protein H-NS (22) and induced by ArcA and FruR (53). In addition, the expression of aerobic respiratory pathways is influenced by iron availability (13) (Figs. 6, 7). Whatever the mechanism, the upregulation of the *cydAB* operon by CO suggests that CO induces a transition to a more anoxic-like state (20).

In contrast, the aerobic downregulation of *cyo* expression is explicable by rising FNR activity alone. Anoxically, *cyo* expression is elevated, but this operon too is under complex regulation, being repressed by ArcA, Fur, and PdhR, as well as FNR. GadE (28) and CRP (75) upregulate *cyo* expression.

### PdhR and other regulators

The *pdhR* gene is the proximal gene of the *pdhR-aceE-aceF-lpd* operon encoding the PDH complex. PdhR functions with other central regulators as an additional master switch (46), negatively regulating expression of the *pdh* promoter, with pyruvate serving as an inducing coeffector (51) and repressing the *cyo* and *ndh* operons (46). The structural genes of the PDH operon (Fig. 2A) as well as *cyoABCD* and *ndh* were all upregulated by CO under anoxic conditions (Fig. 2B and Supplementary Fig. S2), as was the *cyo* operon, implying loss of the repressing activity of PdhR, perhaps due to pyruvate (46). TFInfer analysis (Fig. 4) also shows that aerobically PdhR activity declines, consistent with a CO-induced block of respiration and pyruvate accumulation (Fig. 5D).

Electron flow into the respiratory chains when CO is present is facilitated both aerobically and anoxically (Fig. 2B) by immediate and marked upregulation of *ndh*, encoding NADH:ubiquinone oxidoreductase II, a single-subunit nonproton-translocating enzyme. FNR represses *ndh* expression anoxically and expression of an *ndh-lacZ* fusion in an *fnr* mutant is enhanced by anaerobiosis (23). Therefore, the inferred increase in FNR activity (Fig. 4) and *ndh* upregulation are not consistent with simple repression of *ndh* by FNR. Other regulators may be implicated, such as Fis, which enhances *ndh* expression during rapid growth (31). Expression of Ndh is associated with growth where protonmotive force generation is not favored (10), consistent with the presence of CO. In contrast to *ndh*, the *nuo* genes encoding a multisubunit, proton-pumping NADH dehydrogenase were downregulated (2- to 5 fold) in response to CO in anoxic conditions (Supplementary Fig. S2B). These results together with downregulation of the *cyo* operon (Fig. 2) suggest that CO promotes assembly of a respiratory chain that is not optimized for energy-efficient growth, but for the disposal of reducing equivalents (*i.e.,* NAD(P)H) (10).

### CO exposure perturbs genes involved in amino acid biosynthesis

Global responses to CO included gross alterations in amino acid metabolism (Fig. 1). First, many genes in the *cys* and *tau* operons were upregulated, suggesting a shortage of sulfur in response to CO (Supplementary Fig. S5A). The major regulator is CysB (35), which lies adjacent to FNR in the coherence profile (Fig. 3; bottom left quadrant), indicating that the response was similar in magnitude and kinetics both aerobically and anaerobically. In *Salmonella*, CysB, activated by N-acetylserine in the absence of cysteine (35), forms a DNA-binding tetramer. CysB activates the *cys* regulon for sulfur utilization and uptake, the *tau* operon for taurine utilization, the *cbl* gene encoding a regulator of the sulfate starvation response, and, in coordination with the Cbl protein, the sulfonate utilization genes (*ssuABCDE*) (69). Many of these genes are upregulated in response to CO (Supplementary Fig. S5A) after 10 min of CO exposure, especially *cysB*, *cysC*, *cysD*, *cysH*, and *cysP*, as well as most *tau* genes.

Second, many genes involved in methionine biosynthesis were perturbed by CO (Supplementary Fig. S5B), but with complex patterns of expression. Aerobically, a requirement for methionine appears immediate with many *met* biosynthetic genes upregulated by CO, especially *metA*, *metB*, *metF*, *metK*, *metN*, and *metR.* MetJ, which represses the expression of genes involved in biosynthesis and transport of methionine (5), was elevated in the coherence plot (Fig. 3D), indicating differences in the aerobic and anoxic responses (Supplementary Fig. S5B).

Third, ArgR responded differently to CO aerobically and anoxically (Figs. 3 and Supplementary Fig. S6A). Most arginine biosynthesis genes were highly upregulated aerobically as well as *carA* encoding carbamoyl synthetase. Additionally, arginine transport genes (*artJ-Q*) were upregulated aerobically with *artJ*, encoding the periplasmic protein of the L-arginine transporter, upregulated by 20-fold after 10 min. However, anaerobically, these genes were predominantly downregulated (Supplementary Fig. S6A). Thus, CO appears to lead to arginine deprivation under aerobic conditions. Interestingly, peroxynitrite also increases arginine biosynthesis transcript levels in *E. coli* due to nitration of the ArgR repressor (42). However, CO is relatively inert (CO dehydrogenase being the only documented biological redox activity) and it is unclear how it could modify or degrade ArgR (see the Discussion section).

### CO leads to altered expression of iron acquisition genes

The location of Fur in the upper left quadrant of the coherence plot (Fig. 3A) indicates that it exhibited significantly different responses under aerobic and anoxic conditions. Since the primary biological targets of CO are heme- and [Fe-S] cluster-containing proteins (8), we investigated the role of Fur, the principal TF involved in detecting iron levels and repressing genes for iron acquisition (56). Aerobically (Fig. 6A), the inferred Fur activity initially rose, consistent with downregulation of *ent* genes involved in enterochelin biosynthesis (Figs. 6C and Supplementary Fig. S6B) and the *fep* and *fhu* genes involved in ferric enterobactin transport and hydroxamate-dependent iron uptake, respectively. Anoxically, there was extensive and sustained derepression of iron-regulated genes (Figs. 6D and Supplementary Fig. S6B) and inferred Fur activity fell sharply (Fig. 6B). For example, *entE* (encoding a component of enterobactin synthase) was 30-fold upregulated anaerobically after 10 min; genes involved in enterobactin (*fepABCDG*) and ferrichrome transport (*fhuA-F*) were also upregulated. Overall, the data point to CO-induced iron deprivation, particularly anoxically. The changes in respiratory genes (Fig. 2B and Supplementary Fig. S2B) are also consistent with modulation by iron. Upregulation of *cydAB* expression, particularly anoxically, is consistent with elevated *cydAB* expression by the iron chelator, 2,2′-dipyridyl (13).

### Exposure of cells to CO leads to increased siderophore production and a reduction in the levels of intracellular free iron

Given the changes in *ent* gene expression, it was important to determine whether cell physiology reflected transcript levels. Therefore, cultures growing on Chrome Azurol S (CAS) agar plates were incubated in CO atmospheres. Fe(III) reacts with CAS to produce an intense blue color, but siderophores compete with the dye for Fe(III), causing a color change (blue to orange). Siderophore production on CAS plates caused a clear orange halo around the bacterial growth (not shown). Both aerobically and anaerobically, CO increased halo diameter (corrected for the spot size, representing siderophore production; Fig. 7A), supporting the transcriptomic data, and suggesting that CO leads to iron limitation.

To test whether free intracellular iron pools were depleted upon exposure to CO, EPR analysis of ferric ions in rhombic coordination was performed (34). To measure total free iron, the cell-permeable iron chelator, desferrioxamine (which promotes the oxidation of Fe(II) to Fe(III) and chelates all Fe(III)), was used. Importantly, desferrioxamine does not chelate protein-bound iron. Fe(III) in rhombic coordination gives an EPR signal with *g* = 4.3, which was measured on whole cells (Fig. 7B). Total iron levels decreased by about 50% on exposure to CO both aerobically and anaerobically. Aerobically, free intracellular iron levels are ∼40 μ*M* in the absence of CO, falling to 15 μ*M* with CO (Fig. 7B). As hypothesized, free iron levels decreased anaerobically from ∼200 to ∼100 μ*M* with CO. Thus, CO depletes free cellular iron, consistent with patterns of gene expression and elevated siderophore synthesis.

### CO renders cultures hypersensitive to external chelators

**FIG. 8.**
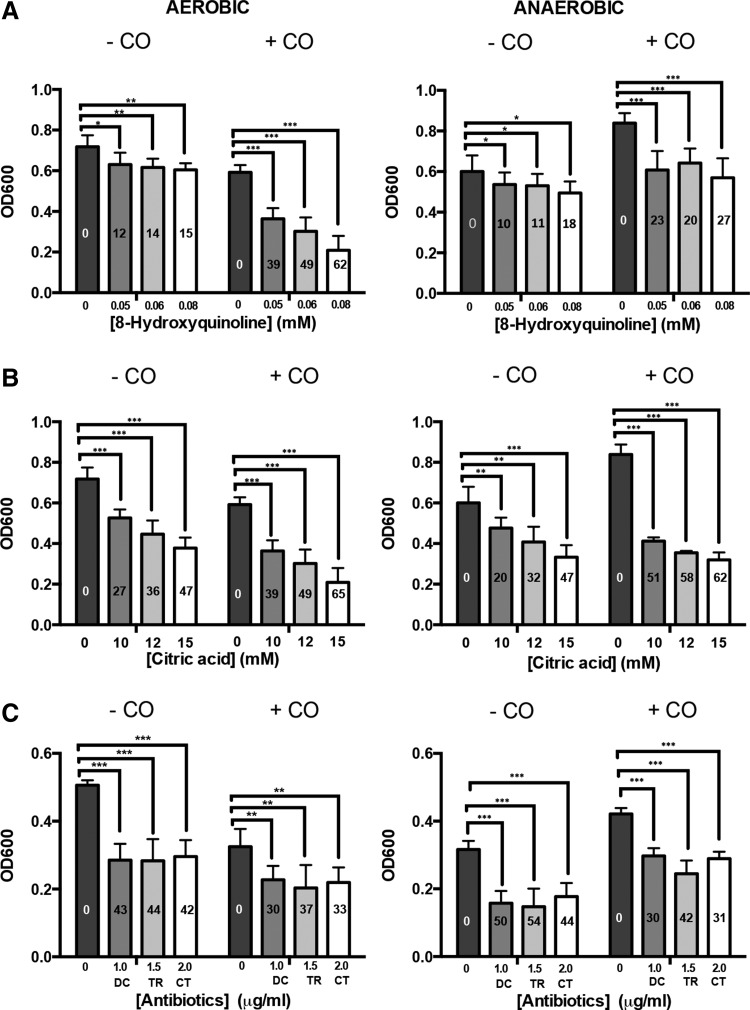
**Effects of CO gas on bacterial sensitivity to metal chelators and antibiotics.** Aliquots of *E. coli* in defined medium were dispensed into the wells of a 96-well plate and supplemented with the following compounds at the concentrations shown: **(A)** 8-hydroxyquinoline, **(B)** citric acid, **(C)** the antibiotics doxycycline (DC), trimethoprim (TR), and cefotaxime (CT). Percentage inhibition was calculated as 100 − (X/Y × 100), where X denotes growth observed in a CO atmosphere (25% CO +75% air or 25% CO +75% N_2_) and Y the observed growth in the equivalent atmosphere without CO. Data are representative of two biological repeats and each of four technical repeats, expressed as mean ± SD. *p < 0.05, **p < 0.01, ***p < 0.001 (Paired *t*-test).

Given the changes in iron-related genes and iron pools, we hypothesized that iron chelators would inhibit growth in CO since intracellular iron limitation would be exacerbated by exogenous chelating activity. Bacteria were therefore grown with 8-hydroxyquinoline or citric acid in CO atmospheres (Fig. 8A, B). For each, the aerobic (left) and anoxic data (right) show the effects of increasing concentrations (as a percentage of the no-chelator control in the same gas atmosphere). The effects of CO (25% by volume of the incubation jar) are compared with the same atmospheres, but in which 25% nitrogen was used as control. CO alone slightly inhibited aerobic growth. 8-Hydroxyquinoline inhibited aerobic growth, relative to the nonchelator control, by up to 62% with CO, but only 15% without (Fig. 8A). Anaerobically, the values were 27% and 18%, respectively. 8-Hydroxyquinoline has a high affinity for Fe(III) (log stability constant = 26.3) and, to a lesser extent, Fe(II) (log stability constant = 15.0) (49), so other chelators with lower specificity were also tested. Cells grown with citric acid (log stability constant with Fe(III) = 11.4) experienced inhibition in growth; anoxically, CO gave up to 65% inhibition of growth relative to the nonchelator control aerobically and up to 62% anaerobically (Fig. 8B). We also performed assays using Biolog phenotype arrays and found that 1,10-phenanthroline (log stability constant with Fe(III) = 14.1) gave similar results to citrate; it inhibited aerobic growth, relative to the nonchelator control, by 78%–88% with CO, but only 37%–81% without. Anaerobically, the values were 54%–87% and 19%–74%, respectively. These data are not shown as the chelator concentrations in proprietary Biolog plates are unknown. We did not use desferrioxamine in these experiments designed to test extracellular retention of iron since this chelator is cell permeable. In conclusion, CO exacerbates inhibition of *E. coli* growth by chelating agents.

### CO and a CORM: antimicrobial agents or protectants against antibiotics?

With the advent of antibiotic resistance, combinatorial approaches may reduce dependency on the use of single antibiotics (45). Both NO and H_2_S protect bacteria from a broad range of antibiotics (24, 59), but CO effects have not been reported. Without antibiotics, CO reduced aerobic growth yield by 36%; anoxically, however, CO stimulated growth by 33% (Fig. 8C). This is shown also in the no-chelator plots in Figure 8A and B, but is currently unexplained. CO only marginally affected growth in the presence of the antibiotics, doxycycline (DC), trimethoprim (TR), or cefotaxime (CT). For example, CT (Fig. 8C) inhibited the no-CO control cells by 42%–44%, but cells grown in a CO-supplemented atmosphere by 31%–33%. The results were similar, irrespective of the presence of oxygen.

Since CO gas is not a potent antimicrobial agent (Supplementary Fig. S1), yet CORMs generally are (70), we compared the effects of CO gas with an extensively used ruthenium CORM, CORM-2. The toxicity of this compound allowed us to use it in agar plates where its effects on the minimal inhibitory concentrations (MICs) of three well-established antibiotics (TR, DC, and CT) were determined. In each case, 30 μ*M* CORM-2 dramatically reduced the MICs, whereas the inactive form of the control compound iCORM-2 (Ru(II)Cl_2_(DMSO)_4_) and the solvent DMSO were without significant effect on the MICs (Supplementary Fig. S7).

## Discussion

CO delivery to key sites offers novel pharmacological and therapeutic approaches, but we know too little of how CO mediates its multiple biological effects. Although CO's affinity for heme proteins is undisputed, its actions *in vivo* are remarkably complex (54), having further roles in NO release, reactive oxygen species formation, and ion channels (54). It has been tacitly assumed that the biological impacts of CORMs are attributable largely to CO delivery to hemes, but microbial (70) and mammalian (54) studies point to greater complexity. Indeed, bacteria are often resistant to CO gas (71), but not to certain CORMs, and demonstrate multiple transcriptomic changes that cannot be understood in terms of known CO biochemistry [*e.g.,* Wilson *et al.* (72)]. Furthermore, even iCORMs, frequently assumed to be CO depleted, can exert toxicity in bacterial systems (41). Most tellingly, cells lacking all hemes are also inhibited by CORM-3 and reveal multiple transcriptomic changes (73). Finally, other compounds of Ru are taken up and have antimicrobial properties, even though they are not CORMs [*e.g.,* Li *et al.* (38)].

We therefore characterized the effects of CO gas *per se*, without mediation of a CORM, on bacterial growth, gene expression, and physiology. HO-derived CO induces the dormancy regulon in *Mycobacterium tuberculosis* (60). The CO-oxidizing Archaeon *Archaeoglobus fulgidus* responds to CO with only limited transcriptional effects (26): of 52 genes observed to alter, inorganic ion transport was the most prominent category, along with *cooF*, encoding a CO dehydrogenase Fe-S enzyme. Bacterial mechanisms for resisting CO are unclear. Zacharia *et al.* (74) used a 2% CO atmosphere to screen for *M. tuberculosis* mutants susceptible to CO, but the resistance test reported for the *cor* gene identified used CORM-2, not CO gas.

**FIG. 9.**
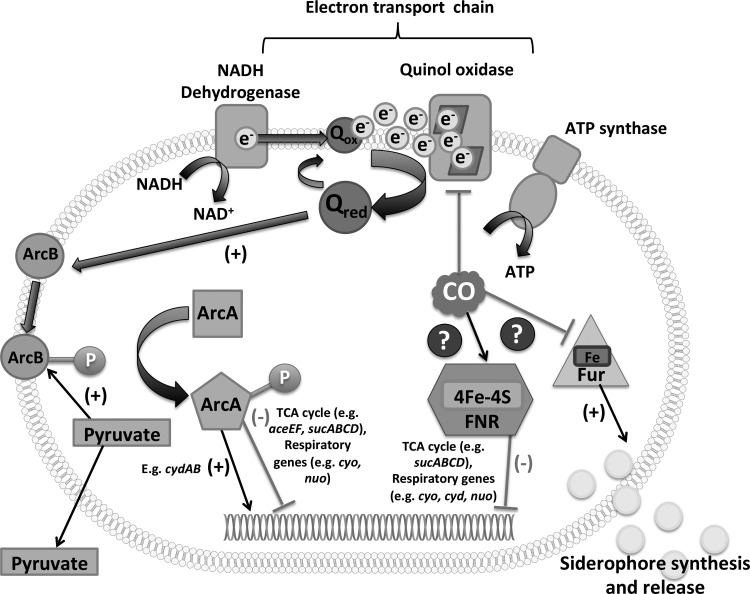
**Schematic diagram of the global impacts of CO gas on** ***E. coli***. Inhibition of the electron transfer chain results in stimuli that cause ArcA phosphorylation, namely over-reduction of the quinone pool and formation of the fermentation product, pyruvate, in concentrations that can be assayed extracellularly. Transcriptomic responses are consistent with direct or indirect modulation by CO of Fnr and Fur activities.

We demonstrate here for the first time that genes encoding energy transduction are significantly affected by CO *via* the master regulators, ArcA and FNR. In the case of Arc, CO inhibition of respiration promotes pyruvate accumulation (Fig. 5D) and may elicit changes in the redox state of the quinone pool (which is not significantly altered in its composition; Supplementary Fig. S3). The effect of CO on UQ reduction levels could be studied in a UQ-only mutant. These changes indirectly result in modulation of Arc activity (6, 29, 31) (Fig. 9). In contrast, FNR is primarily a direct sensor of oxygen, although the 4Fe-4S cluster of the transcriptionally active factor is also sensitive to NO, rendering it inactive as a repressor (15). It is not known whether the Fe-S cluster of FNR is reactive with CO, but CO can bind to other nonheme sites (36, 63).

A striking consequence of CO exposure was enhanced expression of iron acquisition genes and changes in Fur activity. The reason for the loss of total free iron in cells is unclear, but CO may result in more iron being protein bound, perhaps in inactive states. Bacteria thus respond by producing more iron-containing proteins, further draining iron resources. The change in Fur activity may also be due to a direct effect of CO on the protein since the active form of Fur contains a nonheme ferrous iron site with oxygen and nitrogen donor ligands (1). NO reacts with Fe(II) in Fur, inhibiting Fur repressor activity and Fur DNA binding (17), so interaction with other ligands cannot be discounted. Significantly, Fur in *Anabaena* interacts with and binds to heme (61), affecting DNA binding (25). The binding of heme to Fur exhibits the same physicochemical features as heme sensor proteins and the Fe(II) heme-Fur binds CO (48); His is the probable axial ligand trans to CO. Binding of CO to Fur-associated heme would be more likely anoxically, consistent with the upregulation of Fe-related genes under anoxic conditions.

The upregulation of genes involved in amino acid metabolism was unexpected (Supplementary Figs. S5 and S6), although a transcriptomic study with CORM-3 showed similar results, with sulfur metabolism being a prime target (41). The introduction of carbonyl groups into proteins by oxidation of amino acid side chains or by oxidative cleavage of proteins is also possible. Protein carbonylation is metal-catalyzed oxidation, which occurs when reduced metal ions (*e.g.,* Fe(II) or Cu(I)) react with H_2_O_2_ in Fenton chemistry to produce highly reactive hydroxyl radicals. This in turn oxidizes amino acid side chains or causes protein backbone cleavage, both resulting in the formation of carbonyl groups. In bacteria, metal-catalyzed oxidations appear to be the predominant source of carbonylated proteins (44). Arginine is one of the four most commonly carbonylated amino acids. Interestingly, the present data point to iron deprivation on CO treatment and could reflect loss of iron due to carbonylation.

Contrary to recent findings with NO (24) and H_2_S (59), we found no evidence that CO significantly protected bacteria against antibiotics, nor did it potentiate their effect. However, CORM-2, as reported previously for *Helicobacter pylori* (65), markedly reduced the MIC for three commonly used antibiotics. The results imply that the effects of CORMs are not attributed to CO alone and support a role for the metal in CORM toxicity. The mechanisms underlying these effects require urgent investigation to facilitate future applications of combination therapy.

## Materials and Methods

### *E. coli* strains and growth conditions

*E. coli* K12 derivative MG1655 was used throughout. For transcriptomic studies, cells were grown in Evans medium (41) with glucose as the carbon source. In continuous culture, cells were grown in an Infors Multifors bioreactor (working volume 200 ml) adapted to fit a Labfors-3 fermenter base unit. Temperature was maintained at 37°C with continuous stirring at 200 rpm, and pH was maintained at 7.0. Mass flow controllers maintained gas mixes for aerobic and anaerobic conditions by continuous bubbling with air (aerobic) or with N_2_ gas (anaerobic) at 200 ml·min^−1^; CO was bubbled at a rate of 100 ml·min^−1^ with the appropriate gas mix for continuous culture conditions.

### Transcriptomic analysis

Experiments were performed and analyzed as before (41) on samples from chemostat cultures immediately before CO gas addition and at time intervals thereafter. RNA was isolated using phenol–ethanol extraction, and cDNA was synthesized and labeled with Cy-3 or Cy-5-dCTP (41). Arbitrary values of ≥2-fold or ≤0.5-fold changes were chosen to show genes with significantly altered expression. Functional category gene lists were created using KEGG (Kyoto Encyclopedia of Genes and Genomes) (33). Regulatory proteins for each gene were identified using EcoCyc. Modeling of TF activities was carried out using TFInfer (4, 37). Data have been deposited in NCBI's Gene Expression Omnibus and are accessible through GEO series accession number GSE50713 (www.ncbi.nlm.nih.gov/geo/query/acc.cgi?acc=GSE50713).

### Arc phosphorylation assays

For measurements of ArcA phosphorylation, and quinone and pyruvate levels, batch cultures were grown in 200 ml Evans medium (supplemented with 5% LB broth [LB; Miller] for anaerobic growth) in Duran bottles with modified lids allowing gas bubbling into the culture. Cultures were inoculated (5%) with prewashed LB-grown cells and incubated at 37°C in a static water bath with a gas supply of either air (aerobic) or 95% N_2_ with 5% CO_2_ (anaerobic) until an OD_600_ of 0.5–0.6 was reached. Mass flow controllers as above controlled gas mixing and delivery. At time zero, the gas supply to the culture was switched, where appropriate, to a mix of 50% CO and 50% of the original gas. Anaerobic samples were removed using a sterile syringe and needle through a tube without the need to remove the lid.

For measuring ArcA phosphorylation, samples (1 ml) were taken at t = 0 and at 5, 20, 40, and 80 min thereafter and added to a microfuge tube containing 200 μl formic acid (6 *M*). After centrifugation, the pellet was resuspended in 50 μl formic acid (1 *M*), centrifuged again, and the final pellet frozen in liquid nitrogen, then stored at −20°C. ArcA phosphorylation levels were measured using Phos-tag™-acrylamide gel electrophoresis (NARD Institute, Ltd.) and subsequent Western immunoblotting (55). ArcA was immunodetected using rabbit ArcA polyclonal antiserum and the ECL system (GE Healthcare). ArcA protein was purified as before (6). Values were corrected for the OD_600_ of the culture at the point of harvest. ArcA phosphorylation of samples in each of two independent cultures (biological repeats) was analyzed in duplicate and the results of one experiment are shown in Figure 5.

### Quinone extraction and analysis

Culture samples (3 ml) taken at t = 0 and t = 40 min, and immediately frozen in liquid nitrogen, were stored at −20°C. Quinone extraction and measurement methods were adapted from (58). During evaporation of the petroleum ether, 100 μl hexanol was added and the remaining material, dissolved in hexanol, was transferred to HPLC vials and stored at −20°C until analyzed. Standards for UQ, DMK, and MK were prepared from isolated quinone extracts from *E. coli* MG1655. Concentrations for each quinone type were determined using extinction coefficients (27, 43). The quinone content of two independent cultures was analyzed in duplicate.

### Quantification of pyruvate

Cultures were sampled as shown in Figure 5D and the supernatants frozen in liquid nitrogen, then stored at −20°C until the assay was performed. A Pyruvate Assay Kit (MAK071; Sigma Aldrich) was used according to the manufacturer's protocol. Concentrations were corrected for the OD_600_ of the culture at harvest. Pyruvate content of two independent cultures was analyzed in duplicate.

### Siderophore assays

Siderophore production was assayed on CAS agar plates (12) supplemented with a trace element solution that (at 100 × strength) contained (L^−1^) 8 ml concentrated HCl, 0.41 g ZnO, 2 g MnCl_2_.4H_2_O, 0.17 g CuCl_2_.2H_2_O, 0.48 g CoCl_2_.6H_2_O, 64 mg H_3_BO_3_, and 4 mg Na_2_MoO_4_.2H_2_O. Batch cultures were grown in LB medium to an OD_600_ of ∼0.5 and 10 μl portions spotted on 25-ml plates that were incubated for 48 h aerobically (100% air) or in anaerobic jars (HP0011A, Oxoid). A vacuum was first used to withdraw air and the vacuum gauge was read; the first gas (*e.g.,* CO) was then admitted *via* a valve to restore 50% of atmospheric pressure, and then the second gas (*e.g.,* air) was admitted to restore normal atmospheric pressure. Siderophore production was determined by measuring halo diameters (Fig. 7).

### Whole cell electron paramagnetic resonance analysis

The method was derived from (34, 64). Cells were grown in LB to an OD of ∼0.2 before centrifugation at 7800 *g* at room temperature and concentrated 200- to 300-fold before incubation at 37°C for 15 min in 20 m*M* desferrioxamine. Cells were again centrifuged, washed with cold 20 m*M* Tris (pH 7.4), and resuspended in a final volume of ∼0.5 ml Tris/10% glycerol. Aliquots (0.3 ml) were placed in EPR tubes, which were flash-frozen in methanol kept on dry ice. EPR measurements were performed at 10 K on a Bruker EMX EPR spectrometer equipped with a spherical high-quality Bruker resonator SP9703 and an Oxford Instruments liquid helium system for low temperature measurements. To measure intensities of the *g* = 4.3 EPR signal from rhombic ferric iron, the procedure of spectral subtraction with a variable coefficient was used (64).

### Growth of *E. coli* in anaerobic jars to test chelators

Aliquots of a cell suspension (OD 0.05) were dispensed into the wells of a 96-well plate and supplemented with the chelators. Plates were incubated both aerobically (25% CO +75% air or 25% N_2_: 75% air) and anaerobically (25% CO: 75% N_2_ or pure N_2_) in anaerobic jars, as above, at 37°C for 24 h. Preliminary testing of the combined effects of CO and antimicrobial compounds was done using Biolog Phenotype Microarrays.

### Effect of CO gas and CORM-2 on bacterial growth in conjunction with chelators and antibiotics

Aliquots of a cell suspension (OD 0.05) were dispensed into a 96-well plate and supplemented with the following compounds at the concentrations shown in Figures 8 and S7: 8-hydroxyquinoline, citric acid, or the antibiotics DC, TR, and CT. For aerobic experiments, growth in 25% CO +75% air was compared with growth in 25% N_2_ + 75% air. For anaerobic experiments, growth in 25% CO +75% N_2_ was compared with growth in N_2_ alone. Optical density was measured after 24 h of incubation at 37°C. Percentage inhibition was calculated as 100 − (X/Y × 100), where X denotes growth observed in a CO atmosphere (25% CO +75% air or 25% CO +75% N_2_) and Y the observed growth in the equivalent atmosphere without CO.

### Determination of MIC values for antibiotics

Cell suspension (100 μl) was added to 4 ml of 0.5% agar and the mixture poured onto defined minimal agar plates. Plates were left to dry for ∼10 min before antibiotic strips (E-test, bioMérieux) were placed on the surface of the plates, which were then incubated at 37°C for 24 h.

## Supplementary Material

Supplemental data

Supplemental data

Supplemental data

Supplemental data

Supplemental data

Supplemental data

Supplemental data

## Abbreviations Used

ABC: ATP-binding cassette (in ABC transporters)
CAS: Chrome Azurol S
CORM: carbon monoxide-releasing molecule
CORM-2: tricarbonyldichlororuthenium(II) dimer, [Ru(CO)_3_Cl_2_]_2_
CORM-3: Ru(CO)_3_Cl(glycinate)
CT: cefotaxime
DC: doxycycline
DMK: demethylmenaquinone
DMSO: dimethyl sulfoxide
EPR: electron paramagnetic resonance
Fe-S: iron-sulfur (as in cluster)
FNR: fumarate nitrate reduction regulator)
HO: heme oxygenase
iCORM-2: inactivated CORM-2
MIC: minimal inhibitory concentration
MK: menaquinone
OD: optical density
PDH: pyruvate dehydrogenase
TCA: tricarboxylic acid
TF: transcription factor
TR: trimethoprim
UQ: ubiquinone
