# Supplementary material for: Carbon Monoxide Gas Is Not Inert, but Global, in Its Consequences for Bacterial Gene Expression, Iron Acquisition, and Antibiotic Resistance
Source: Antioxid Redox Signal. 2016 Jun 10;24(17):1013–28. doi: 10.1089/ars.2015.6501 (PMC4921903; doi:10.1089/ars.2015.6501)
Supplement: Supplemental data [file Supp_Fig1.pdf]

Supplementary Data

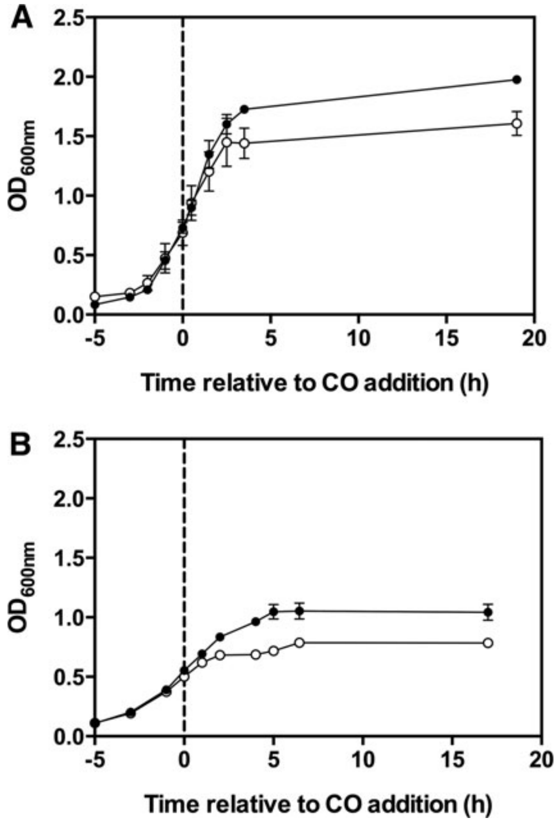

SUPPLEMENTARY FIG. S1. Growth of *Escherichia coli* cells aerobically and anaerobically in the presence of CO. *E. coli* MG1655 cells were grown as batch cultures in the fermenter vessel under aerobic **(A)** or anoxic conditions **(B)** until an OD of ~0.5 was reached. CO was switched on at 100 ml·min^{-1} at the *dashed line*. Results plotted are of two biological repeats ± SDs. OD, optical density.
