# Supplementary material for: Carbon Monoxide Gas Is Not Inert, but Global, in Its Consequences for Bacterial Gene Expression, Iron Acquisition, and Antibiotic Resistance
Source: Antioxid Redox Signal. 2016 Jun 10;24(17):1013–28. doi: 10.1089/ars.2015.6501 (PMC4921903; doi:10.1089/ars.2015.6501)
Supplement: Supplemental data [file Supp_Fig3.pdf]

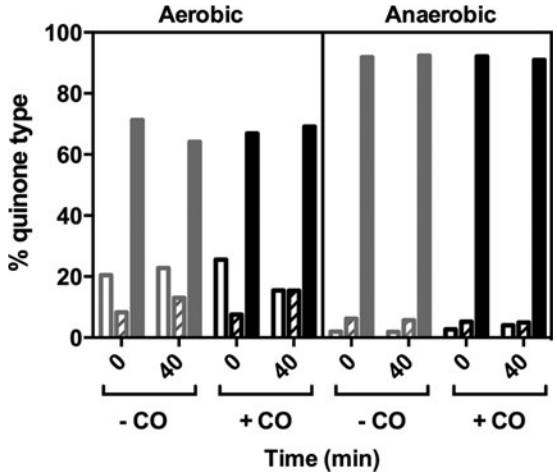

SUPPLEMENTARY FIG. S3. Quinone species in cells exposed to CO. Quinones were extracted from cell pellets at t = 0 and t = 40 min after CO bubbling and assayed by HPLC. In each condition, UQ is shown by *open bars*, DMK by *hatched bars*, and MK by *solid bars*. DMK, demethylmenaquinone; MK, menaquinone; UQ, ubiquinone.
