# Supplementary material for: Carbon Monoxide Gas Is Not Inert, but Global, in Its Consequences for Bacterial Gene Expression, Iron Acquisition, and Antibiotic Resistance
Source: Antioxid Redox Signal. 2016 Jun 10;24(17):1013–28. doi: 10.1089/ars.2015.6501 (PMC4921903; doi:10.1089/ars.2015.6501)
Supplement: Supplemental data [file Supp_Fig4.pdf]

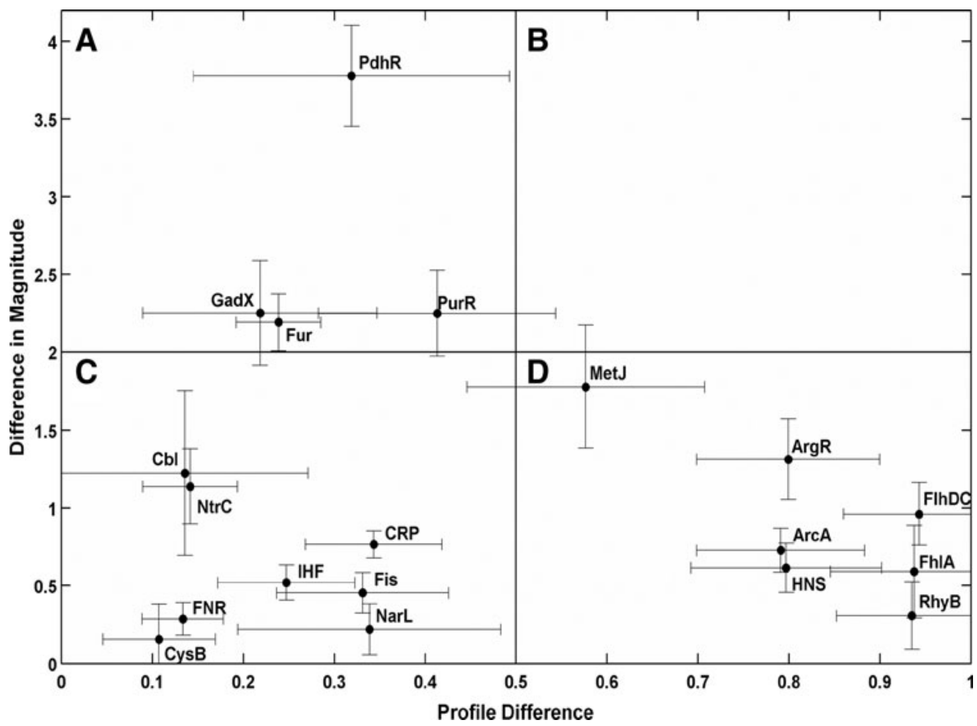

SUPPLEMENTARY FIG. S4. Coherence plot showing TFs involved in the response to CO gas aerobically *versus* anaerobically. The same data as in Supplementary Figure S3 are shown here, but with error bars (1.96 times standard deviation or 95% confidence interval) for the profile difference and difference in magnitude, to demonstrate the uncertainty in these measurements, arising from the uncertainty of the inferred TF profiles derived from TFInfer. TF, transcription factor.
