# Supplementary material for: Carbon Monoxide Gas Is Not Inert, but Global, in Its Consequences for Bacterial Gene Expression, Iron Acquisition, and Antibiotic Resistance
Source: Antioxid Redox Signal. 2016 Jun 10;24(17):1013–28. doi: 10.1089/ars.2015.6501 (PMC4921903; doi:10.1089/ars.2015.6501)
Supplement: Supplemental data [file Supp_Fig5.pdf]

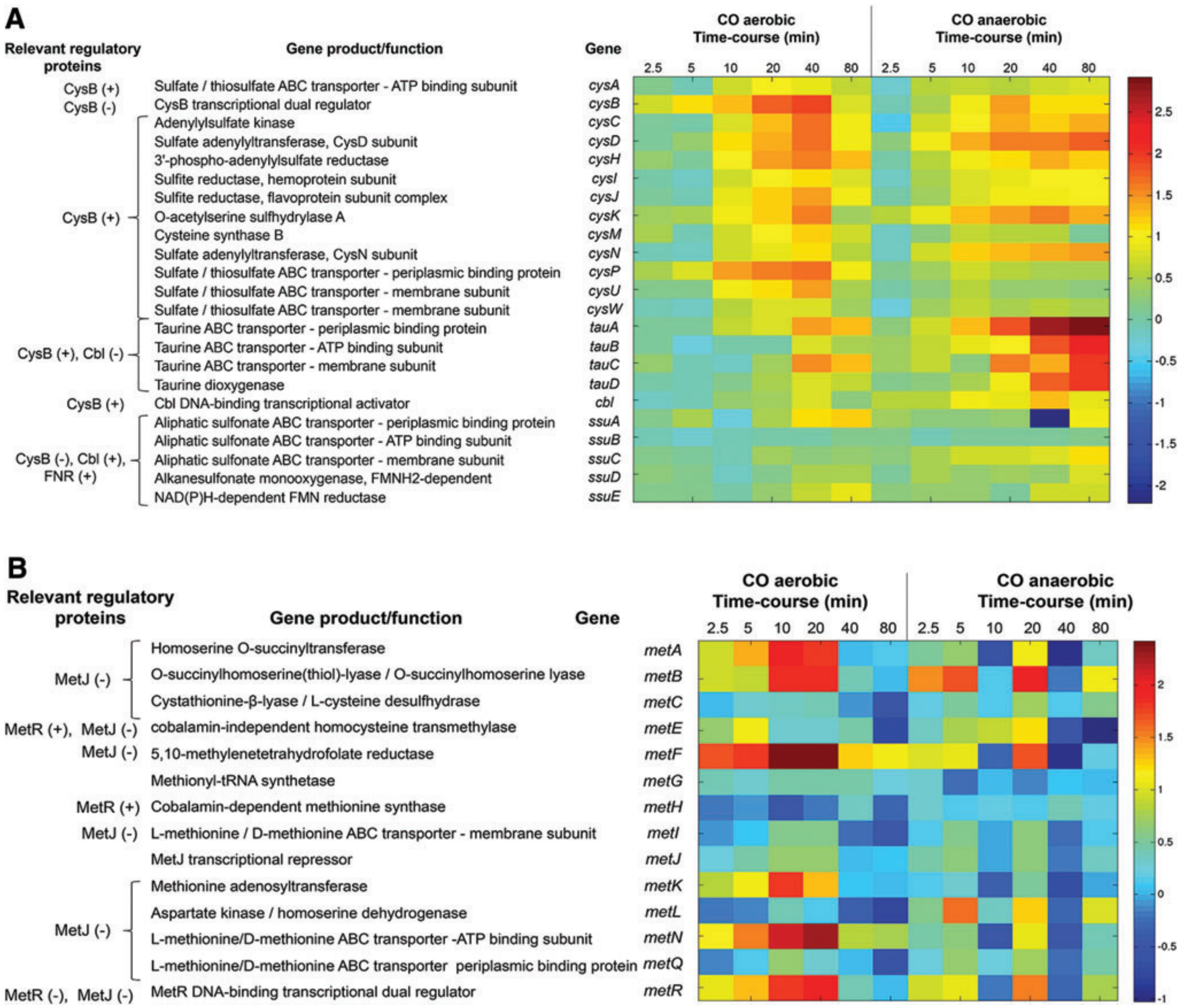


**SUPPLEMENTARY FIG. S5. Differential expression of genes involved in cysteine uptake and metabolism (A) and methionine biosynthesis and transport (B).** The heat map quantifies the changes in selected genes; note that the heat scale at the right is expressed as the natural logarithm of the fold change in genes. *E. coli* cells were grown aerobically and anaerobically in Evans medium with glucose after addition of CO gas at 100 ml·min$^{-1}$.
