# Supplementary material for: Carbon Monoxide Gas Is Not Inert, but Global, in Its Consequences for Bacterial Gene Expression, Iron Acquisition, and Antibiotic Resistance
Source: Antioxid Redox Signal. 2016 Jun 10;24(17):1013–28. doi: 10.1089/ars.2015.6501 (PMC4921903; doi:10.1089/ars.2015.6501)
Supplement: Supplemental data [file Supp_Fig7.pdf]

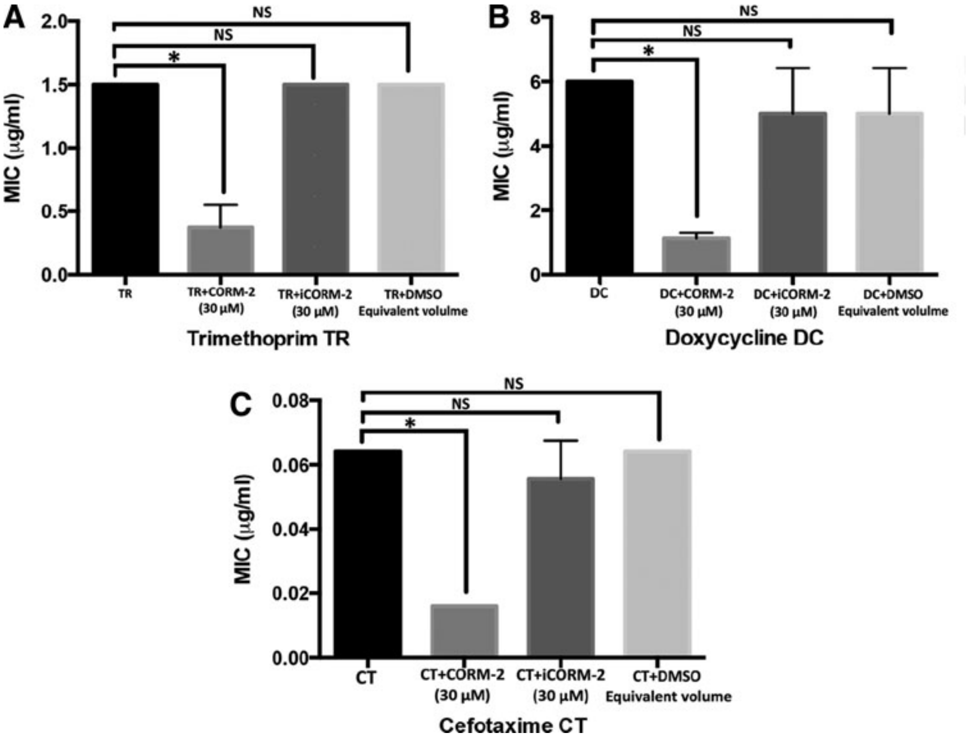

SUPPLEMENTARY FIG. S7. CORM-2 potentiates the effects on *E. coli* of three antibiotics: trimethoprim (TR), doxycycline (DC), and cefotaxime (CT). MICs were determined after 24 h of incubation at 37°C in the presence of 30 μM CORM-2, Ru(II)Cl_2(DMSO)$_4$ (as inactivated or iCORM-2), or an equivalent volume of DMSO. Results show the action of antibiotics only (*black bar*), antibiotic in combination with 30 μM CORM-2 (*light gray*), in combination with 30 μM iCORM-2 (*dark gray*), or with the equivalent volume of DMSO (*very light gray*) on MIC values. Data are representative of two biological repeats and expressed as mean ± SD. NS, nonsignificant; $*p < 0.05$ (Paired *t*-test). CORM, carbon monoxide-releasing molecule; MIC, minimal inhibitory concentration.
